# Multi‐omics QTL provide insights into cell specific effects on molecular functions and intermediate traits in Alzheimer’s disease and its iPSC‐derived model systems

**DOI:** 10.1002/alz.092050

**Published:** 2025-01-03

**Authors:** Philip L. De Jager, Gao Wang

**Affiliations:** ^1^ Columbia University Irving Medical Center and the Taub Institute for Research on Alzheimer’s Disease and the Aging Brain, New York, NY USA; ^2^ Columbia University, New York, NY USA

## Abstract

**Background:**

We examined AD‐associated loci to demonstrate how the new FunGen‐xQTL resource reveals new insights into the sequence of events leading from health to the amyloid and tau proteinopathies that define AD, as well as subsequent cognitive decline.

**Method:**

We utilized FunGen‐xQTL resources (including cell subtype‐specific eQTL results) to deconstruct the genetic regulation and cellular specificity of AD loci. Using transcriptomic and proteomic data systematically derived from iPSC‐derived neurons and astrocytes in up to 48 iPSC lines we highlight and further dissect those genetic effects that replicate in the proper induced iPSC‐derived neuron (iN) or astrocyte (iAstro) model system.

**Result:**

We will discuss illustrative results from our analyses, such as an interaction of CD33 and TREM2: the CD33 risk allele influences the abundance of the CD33 protein isoform and also affects the abundance of TREM2 protein in trans on the same cell surface. Further, in the APOE locus, the e2, e3, e4 haplotypes influence AD risk but not gene expression. We discovered a novel microglial‐specific eQTL for APOE driven by a variant that is not in linkage disequilibrium with APOEe4. Unlike APOEe4, this variant is not associated with AD susceptibility, but it is associated with cerebral amyloid angiopathy (CAA). Thus, APOE expressed by microglia is implicated in the emergence of CAA. Finally, we find that many neuron‐ and astrocyte‐specific QTLs discovered in single nucleus‐derived brain data, replicate in iN and iAstro. However, some eQTLs ‐ such as one affecting MAPT ‐ were significant in both datasets but had an opposite effect size in the iAstro/iN vs. the brain.

**Conclusion:**

Our findings highlight how the FunGen xQTL integrated resource facilitates the discovery and elaboration of the causal chain linking a genetic variant to a series of molecular phenotypes and ultimately a clinical syndrome. We will gradually expand this foundation to map how AD loci interact with one another to perturb the molecular state of the brain and eventually lead to cognitive decline.

